# Natural products: potential treatments for cisplatin-induced nephrotoxicity

**DOI:** 10.1038/s41401-021-00620-9

**Published:** 2021-03-09

**Authors:** Chun-yan Fang, Da-yong Lou, Li-qin Zhou, Jin-cheng Wang, Bo Yang, Qiao-jun He, Jia-jia Wang, Qin-jie Weng

**Affiliations:** 1 Center for Drug Safety Evaluation and Research, College of Pharmaceutical Sciences, Zhejiang University, Hangzhou, 310058 China; 2 Medication Department, Zhuji People’s Hospital of Zhejiang Province, Zhuji, 311800 China

**Keywords:** natural products, cisplatin, acute kidney injury, nephrotoxicity, antioxidant, anti-inflammation, anti-apoptosis, nephroprotection

## Abstract

Cisplatin is a clinically advanced and highly effective anticancer drug used in the treatment of a wide variety of malignancies, such as head and neck, lung, testis, ovary, breast cancer, etc. However, it has only a limited use in clinical practice due to its severe adverse effects, particularly nephrotoxicity; 20%–35% of patients develop acute kidney injury (AKI) after cisplatin administration. The nephrotoxic effect of cisplatin is cumulative and dose dependent and often necessitates dose reduction or withdrawal. Recurrent episodes of AKI result in impaired renal tubular function and acute renal failure, chronic kidney disease, uremia, and hypertensive nephropathy. The pathophysiology of cisplatin-induced AKI involves proximal tubular injury, apoptosis, oxidative stress, inflammation, and vascular injury in the kidneys. At present, there are no effective drugs or methods for cisplatin-induced kidney injury. Recent in vitro and in vivo studies show that numerous natural products (flavonoids, saponins, alkaloids, polysaccharide, phenylpropanoids, etc.) have specific antioxidant, anti-inflammatory, and anti-apoptotic properties that regulate the pathways associated with cisplatin-induced kidney damage. In this review we describe the molecular mechanisms of cisplatin-induced nephrotoxicity and summarize recent findings in the field of natural products that undermine these mechanisms to protect against cisplatin-induced kidney damage and provide potential strategies for AKI treatment.

## Introduction

Cisplatin is a clinically advanced and highly effective anticancer drug that is used for the treatment of various solid tumors, such as lung cancer, stomach cancer, and ovarian cancer [1]. However, nephrotoxicity is the major side effect of cisplatin administration. Clinically, the risk of nephrotoxicity in patients taking cisplatin is between 20% and 35% and leads to death in acute kidney injury (AKI) patients [2, 3]. In addition, pediatric patients also develop nephrotoxicity when using cisplatin [4]. Patients with AKI are clinically characterized by impaired renal tubular function, acute renal failure, a reduction in whole blood cells, anemia, physical tremors, weight loss, gastrointestinal dysfunction, lethargy, and orbital tightening, which limit the antitumor use of cisplatin [5]. Cisplatin mediates nephrotoxicity via a number of different cytotoxic mechanisms. In addition to DNA damage, cisplatin also causes cytoplasmic organelle dysfunction, particularly in the endoplasmic reticulum and mitochondria, activates apoptotic pathways, and inflicts cellular damage via oxidative stress and inflammation [6].

Presently, there is no clinically effective drug to prevent or treat cisplatin-induced nephrotoxicity. Many high-efficacy and low-toxicity drugs from natural products have been developed to protect against cisplatin-induced AKI. For example, ginseng, curcumin, and pomegranate can act as antioxidants and anti-inflammatory agents and possibly protect against oxidative stress by restoring the levels of antioxidant enzymes [7]. In addition, pretreatment with vitamin supplements, such as vitamin E and riboflavin (vitamin B), significantly reduces serum urea and increases the expression levels of antioxidant enzymes in children with steroid-responsive nephrotic syndrome [8]. These natural products have potential antioxidant and anti-inflammatory properties and can be used as supplements to alleviate cisplatin-induced nephrotoxicity.

In this review, we first introduce the pathological manifestations of cisplatin-induced nephrotoxicity and clarify the molecular events of the underlying mechanisms. Finally, we summarize the roles of various kinds of natural products in protecting against cisplatin-induced AKI. This review focuses on the different mechanisms and protective effects of natural products, providing a comprehensive understanding of the prevention of cisplatin-induced nephrotoxicity and potential implications for drug combinations or natural supplements for AKI patients.

## Pathological manifestations of cisplatin-induced nephrotoxicity

Clinically, different doses of cisplatin may lead to different degrees of nephrotoxicity. Patients who receive a single dose of cisplatin may suffer from reversible kidney injury, while large doses or multiple courses of treatment may cause irreversible renal failure [9]. Pharmacokinetic studies also show that nephrotoxicity is mainly due to the high volume of cisplatin distribution and long-term accumulation of cisplatin in the kidney [10]. In general, the pathological mechanisms of cisplatin-induced nephrotoxicity mainly manifest as decreases in renal blood flow and glomerular filtration rate [11] and ischemia or necrosis of proximal renal tubular epithelial cells [12].

**Fig. 1:**
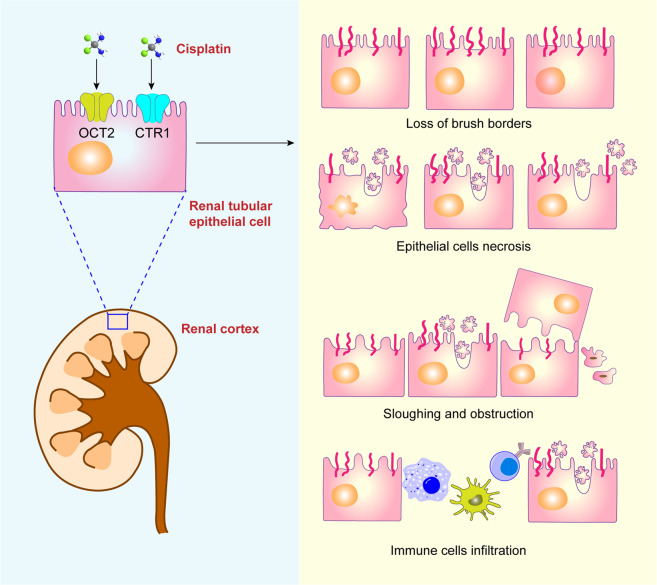
Schematic illustration of pathological manifestations of cisplatin-induced nephrotoxicity. The normal epithelium is damaged by cisplatin, as characterized by the loss of brush borders, epithelial cell necrosis, sloughing and obstruction, and immune cell infiltration.

Histopathological changes in cisplatin-induced nephrotoxicity are positively correlated with the dose of cisplatin. First, cisplatin is passively absorbed into renal tubular cells via organic cation transporter 2 (OCT2) and forms hydrates with water molecules, leading to continuous accumulation in renal cells [13]. The formation of cisplatin hydrate is a reversible process, and cisplatin hydrate can be dissociated into cisplatin and water molecules and discharged from the cells [13]. Thus, the accumulation and retention of cisplatin in renal cells leads to DNA damage, oxidative stress, apoptosis, and autophagy (Fig. 1).

Cisplatin first causes shedding of the brush shape of renal tubular epithelial cells. With increasing cisplatin accumulation, epithelial cells undergo necrosis and are gradually shed, accompanied by the formation of proteinaceous casts [14]. Moreover, the proximal tubule basement membrane becomes thickened, and tubules become dilated [15]. Electron microscopy observation of epithelial cell ultrastructure shows swollen and vacuolated mitochondria, endoplasmic reticulum expansion, and increased numbers of lysosomes [16]. Taken together, these organelle malfunctions result in the destruction and sloughing of epithelial cells, as well as the formation of intratubular obstructions.

Damaged renal tubular epithelial cells recruit many immune cells, such as macrophages, dendritic cells, and T cells, which release a variety of inflammatory factors [17]. Moreover, cisplatin can cause reduced medullary blood flow and exacerbate tubular cell injury, leading to acute ischemic injury in the kidneys [18]. Instead of the typical self-regulatory renal vasodilation in ischemic kidneys, evident vasoconstriction occurs in cisplatin-induced AKI, leading to hypoxic injury and vascular injury in severe cases [19]. Some studies have shown that cisplatin forms a complex with reduced glutathione in the liver and then enters the kidney. Cisplatin is decomposed into a nephrotoxic metabolite due to the action of glutamyltransferase in the brush edge of the renal proximal tubule, causing renal cell apoptosis or necrosis [20].

## Mechanisms of cisplatin-induced nephrotoxicity

**Fig. 2:**
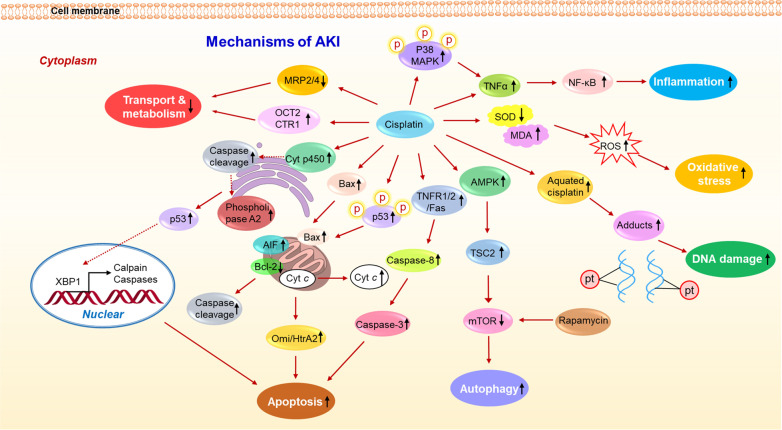
The mechanism summary of cisplatin-induced nephrotoxicity. The mechanisms mainly include the transport and metabolism of cisplatin, apoptosis, autophagy, DNA damage, oxidative stress, and inflammation, which work together to aggravate AKI induced by cisplatin.

The application of cisplatin chemotherapy is often limited by severe adverse effects, including nephrotoxicity, ototoxicity, neurotoxicity, and vomiting. Nephrotoxicity, which is the major limiting factor of cisplatin use, involves various mechanisms, such as oxidative stress, apoptosis, inflammation, and autophagy (Fig. 2). Understanding the underlying mechanism is important for investigating intervention strategies for nephrotoxicity.

### Cellular uptake and transport

Cisplatin is mainly excreted through the kidneys. It becomes concentrated during excretion, and the concentration in renal tubular epithelial cells is much higher than that in the blood. In the kidney, cisplatin is absorbed by renal cells via passive diffusion. During excretion, cisplatin and its metabolites are secreted and reabsorbed in the renal tubules during glomerular filtration, leading to a high concentration of cisplatin in the kidneys.

Recent studies have shown that cisplatin is taken up by renal tubular cells via OCT2, copper ion transporter 1 (CTR1), and solute carrier family 22 member 2 [21]. In addition, cisplatin is secreted into the lumen by solute carrier family 47 member 1 and multidrug and toxin extrusion 1 [22]. Knockdown of the *Oct2* gene can significantly reduce cisplatin-induced nephrotoxicity [23]. Consistently, patients with *Oct2* mutations show low OCT2 expression and reduced cisplatin transport into renal tubular cells, resulting in decreased nephrotoxicity [24]. In addition, when CTR1 expression is downregulated, cisplatin uptake and the subsequent cytotoxicity decrease significantly [25]. Moreover, peroxiredoxin I (*Prx I*)-deficient mice have higher resistance to cisplatin-induced nephrotoxicity than wild-type mice due to increased cisplatin excretion via the high expression of the renal efflux transporters multidrug resistance-related protein 2 (MRP2) and MRP4 in *Prx I*-deficient mice [26].

### DNA damage

Cisplatin mediates its cytotoxic effects by binding DNA to form adducts that cause DNA damage [27]. In an aqueous environment, the chloride ligand of cisplatin is replaced by water molecules to form a positively charged hydrated complex ion, which is transferred to the nucleus by DNA electrostatic attraction. Then, this complex binds to DNA to form an adduct, resulting in DNA cross-linking and preventing DNA synthesis and replication in rapidly proliferating cells [28]. This phenomenon is pronounced in cells with defective DNA repair.

However, cisplatin binds nonspecifically to nuclear DNA, and less than 1% of platinum binds to nuclear DNA [29]. Interestingly, mitochondrial DNA is more sensitive than nuclear DNA to cisplatin-mediated cytotoxicity [30]. The positively charged metabolites produced by the hydrolysis of cisplatin preferentially accumulate in mitochondria, which are negatively charged. Therefore, the sensitivity of cells to cisplatin depends on mitochondrial density and the mitochondrial membrane potential in cells [31]. Given that the renal proximal tubule contains sites of quite high mitochondrial density, it is the most highly sensitive site in the kidney to cisplatin [32].

### Apoptosis

It has been reported that a low concentration (8 μM) of cisplatin causes renal tubular epithelial apoptosis, while a high concentration (800 μM) of cisplatin induces necrosis [33]. Cisplatin-induced apoptosis in renal tubular cells is primarily associated with mitochondria-mediated endogenous pathways, death receptor-mediated exogenous pathways, and endoplasmic reticulum stress (ERS) pathways.

#### Mitochondria-mediated endogenous pathways

Cisplatin-induced mitochondria-mediated apoptotic pathways mainly include caspase-dependent and -independent pathways. When cisplatin enters renal tubular epithelial cells, BAX translocates to mitochondria and activates caspase-2, resulting in the release of cytochrome *c*, second mitochondria-derived activator of caspase/direct inhibitor of apoptosis proteins binding protein with low Pi (isoelectric point) (SMAC/DIABLO), high temperature requirement A2 (HtrA2/Omi), and apoptosis-inducing factor (AIF) from mitochondria [34]. Then, caspase-9 is activated, which eventually leads to apoptosis [35]. Apart from the caspase-dependent pathway, cytoplasmic Omi/HtrA2 also promotes caspase-independent apoptosis by binding and cleaving inhibitors of apoptotic proteins after cisplatin-induced apoptotic stimulation [36].

AIF is an apoptosis-related protein located on the mitochondrial membrane, and poly (ADP-ribose) polymerase-1 (PARP-1) is a nuclear factor that participates in DNA repair and protein modification. Once cellular DNA is severely damaged by cisplatin, nuclear PARP-1 activity is increased, causing AIF activation and nuclear translocation, which induces apoptosis [37]. PARP-1 activation is a primary signal in the process of cisplatin-induced nephrotoxicity. Moreover, PARP-1 inhibition or deletion protects the kidneys from nephrotoxicity, providing a therapeutic strategy for cisplatin-induced nephrotoxicity [38].

The role of p53 in cisplatin-induced cytotoxicity mainly involves activation of the mitochondrial pathway. Upon exposure to cisplatin-induced cellular DNA damage, p53 is phosphorylated, and the proapoptotic protein BAX undergoes structural modifications and alters mitochondrial membrane integrity, causing the activation of p53 upregulated modulator of apoptosis-α and Ca^2+^-independent phospholipase A2. Then, the antiapoptotic proteins BCL-2 and BCL-XL are downregulated, triggering the mitochondrial apoptotic pathway [39].

#### Death receptor-mediated exogenous pathways

In the exogenous apoptotic pathways, cisplatin binds to death receptors such as tumor necrosis factor receptor 1 (TNFR1), TNFR2, and FAS on the cell membrane to activate caspase-8, which further activates caspase-3, ultimately leading to apoptosis [40]. Cisplatin upregulates the expression of tumor necrosis factor-α (TNF-α), promoting the interaction of TNF-α and TNF receptors, including TNFR1 and TNFR2. TNFR1 has a death domain and is able to directly trigger exogenous apoptosis. However, TNFR2 mainly regulates the inflammatory response to induce apoptosis because it has no death domain [41]. In addition, cisplatin can also activate the FAS/FAS-L system [42], and the FAS-associated death domain further interacts with FAS or TNFR1 to trigger apoptosis, but the detailed mechanisms have not been elucidated.

#### Endoplasmic reticulum stress pathways

Cisplatin can also activate the apoptotic pathway that is mediated by ERS. After cisplatin enters cells, it acts on the cytochrome P450 (CYP450) enzymatic system on the endoplasmic reticulum membrane to induce oxidative stress and activate caspase-12, which leads to apoptosis [43]. As expected, cisplatin-induced apoptosis is significantly reduced in cytochrome P450, family 2, subfamily E, polypeptide 1 (*Cyp2e1*)-knockout mice [44]. Similarly, another study showed that the expression of the ERS marker X-box-binding protein 1 was increased, and calpain and caspase-12 cleavage products were observed in rat kidneys after cisplatin treatment [45]. Furthermore, transfection with an anti-caspase-12 antibody significantly attenuated cisplatin-induced apoptosis in porcine kidney LLC-PK1 cells [46]. The ERS pathway is also involved in the activation of endoplasmic reticulum phospholipase A2, which limits downstream p53 and activates upstream caspase-3. The endoplasmic reticulum may be a link between p53 and caspase-3 in the absence of mitochondrial dysfunction [47].

### Oxidative stress

In recent years, studies have shown that oxidative stress and nitrosative stress play vital roles in cisplatin-induced nephrotoxicity, which is characterized by increased malondialdehyde (MDA), 4-hydroxy, 8-hydroxydeoxyguanosine, and 3-nitrotyrosine, and decreased superoxide dismutase (SOD) and catalase (CAT) after cisplatin treatment. Thus, reactive oxygen species (ROS) scavengers and antioxidants show robust protective effects against nephrotoxicity [48].

After entering renal tubular cells, cisplatin can rapidly react with the thiol-containing antioxidants glutathione and metallothionein to degrade or inactivate them. Moreover, some antioxidant enzymes, such as glutathione peroxidase, SOD, and glutathione reductase, are also inhibited, leading to increased ROS levels [49]. ROS affect the activity of mitochondrial complex enzymes I–IV, thereby inhibiting the normal transmission of the oxidative respiratory chain and leading to adenosine triphosphate depletion [40]. Then, increased ROS results in lipid peroxidation, changing membrane structure and permeability, which further affect cellular function [50]. Finally, ROS impair amino acids, proteins, and carbohydrates, thus promoting DNA damage and apoptosis. In addition, increased ROS can induce increased expression of FAS-L, FAS, TNFR1, and TNF-α, eventually resulting in apoptosis [45].

### Inflammation

Cisplatin-induced nephrotoxicity is associated with the inflammatory response. Renal TNF-α expression is increased in a cisplatin-induced nephrotoxic mouse, and cisplatin-induced renal insufficiency and injury can be significantly alleviated by TNF-α inhibition or knockout, indicating that increased TNF-α expression plays an important role in cisplatin-induced nephrotoxicity [51]. Interestingly, after cisplatin administration, TNF-α in the circulation and urine may be derived from renal epithelial cells rather than immune cells. Moreover, TNF-α induces the production of ROS, further activating the transcription factor, nuclear factor kappa-light-chain-enhancer of activated B cells (NF-κB), which in turn induces the production of proinflammatory cytokines such as TNF-α [52]. The inhibition of NF-κB transcriptional activity by JSH-23 (a kind of NF-κB inhibitor) improves kidney function in mice [53].

TNF-α activates proinflammatory cytokines and chemokines to trigger oxidative stress, ultimately exacerbating kidney damage. Hydroxyl free radicals produced by cisplatin are involved in the phosphorylation of p38 mitogen-activated protein kinase (p38 MAPK) and the regulation of TNF-α synthesis, ultimately inducing the activation of NF-κB. Therefore, the hydroxyl radical scavenger dimethyl thiourea inhibits p38 MAPK activation and TNF-α mRNA expression in murine kidneys. The inhibition of p38 MAPK reduces the production of TNF-α, thereby effectively protecting against cisplatin-induced kidney damage [54]. Other cytokines, such as transforming growth factor-β, monocyte chemoattractant protein-1 (MCP-1), intercellular adhesion molecule, and heme oxygenase-1 (HO-1), are also associated with cisplatin-induced nephrotoxicity [55]. N-Acetylcysteine (NAC), an antioxidative agent, effectively inhibits inflammation and activation of the complement system to exert renal protection [56]. Mitochondrial dysfunction leads to the formation of O^2–^, while the inflammatory response induced by cisplatin involves the upregulation of TNF-α, nicotinamide adenine dinucleotide phosphate oxidase, and inducible nitric oxide synthase (iNOS), which directly leads to NO^–^ formation. NO^–^ and O^2–^ produce ONOO^–^, which has strong oxidation and nitration properties, further inducing apoptosis and necrosis [57].

### Autophagy

Autophagy plays an important role in maintaining cellular homeostasis and surviving cisplatin-induced nephrotoxicity. In NRK-52E cells treated with cisplatin, the increases in autophagy and apoptosis were both inhibited after beclin-1 knockdown, indicating that autophagy mediates cell damage [58]. However, another study showed that autophagy inhibition accelerated apoptosis, demonstrating the protective effect of autophagy in cisplatin-induced kidney injury [59]. Moreover, autophagy can prevent AKI and proximal tubule apoptosis caused by cisplatin [60].

Studies have reported that the suppression of mammalian target of the rapamycin (mTOR) activity alleviates the inhibitory phosphorylation of Unc-51-like autophagy activating kinase 1, which leads to the activation of autophagy [61]. Pretreatment with rapamycin, an mTOR inhibitor, induces autophagy to improve renal function in rats with ischemia/reperfusion [62]. Interestingly, NAD(P)H quinone dehydrogenase 1 deletion (an oxidative stress barrier) enhances the effect of rapamycin and leads to increased tuberous sclerosis complex 2 phosphorylation, indicating that autophagy may be activated to counter the increased stress and protect against AKI [63].

## Current treatment of cisplatin-induced nephrotoxicity

Various treatments have been applied to address the different mechanisms of cisplatin-induced nephrotoxicity (Table 1). For example, cimetidine acts as an OCT2 inhibitor that inhibits the transportation of cisplatin in the kidney to protect against AKI [64], carvedilol works as an antioxidant against the oxidative stress process [65], cilastatin inhibits the apoptotic pathway [66], and rosiglitazone reduces inflammation [67].

**Table 1:** Current treatments for cisplatin-induced nephrotoxicity.

Strategies	Mechanisms	Advantages	Limitations
Cimetidine	OCT2 inhibitor [64]	Treat gastric ulcer and gastrointestinal bleeding	May induce mental confusion, hematologic depression, cardiac depression, and hypersensitivity-type hepatitis in elderly patients, patients with nephropathy or liver disease [159]
Carvedilol	Antioxidant, inhibit oxidative stress process [65]	Treat hypertension	Cause liver damage and peripheral vascular disease
Cilastatin	Anti-apoptosis [67]	Protect cyclosporin A-induced nephrotoxicity in clinic [160], septicemia, peritonitis infection, etc.	Cause gastrointestinal adverse reactions, skin allergic reactions, hepatorenal toxicity
Rosiglitazone	Anti-inflammation [66]	Treat diabetes and complications, improve blood lipid level	In treatment of tumors, only for ovarian cancer, limited in other tumors
Amifostine	Cytoprotective agent [70]	Reduce nephrotoxicity and neurotoxicity; the only available therapy that can ameliorate the cumulative nephrotoxic effects of cisplatin without reducing antitumor efficacy [70].	Blood pressure drops and hypocalcemia
Hydration and diuresis, with magnesium or mannitol supplementation	Enhance cisplatin excretion and reduce renal exposure	Safe and feasible, convenient to patients [161]	A large amount of hydration, long cycle period
Chemoprevention (e.g., sodium thiosulfate)	Chemical action (reduction reaction)	Detoxication is quite effective	Osmotic pressure change
Hemodialysis	Principle of material exchange	Removes metabolites and regulates electrolyte and acid-base balance	Hypophosphatemia and heart rate disorder, high cost

At present, although several kinds of drugs are applied clinically in response to kidney damage caused by cisplatin, these drugs exhibit different degrees of inadequacy. For example, hydration and diuresis in the clinic enhance cisplatin excretion and reduce renal exposure [68]. However, the disadvantage is that a large amount of hydration is required before and after cisplatin administration [69]. Moreover, adverse reactions such as osmotic pressure changes may occur during chemoprevention. In addition, metabolic waste in the body can be excreted through hemodialysis, which is often accompanied by hypophosphatemia and heart rate disorders. Amifostine is a broad-spectrum cytoprotective agent approved by the FDA as a kidney protectant for cisplatin chemotherapy in patients with advanced ovarian cancer; however, its application in other tumors is limited due to blood pressure drops and hypocalcemia [70].

## Protective effects of natural products that prevent cisplatin-induced nephrotoxicity

**Fig. 3:**
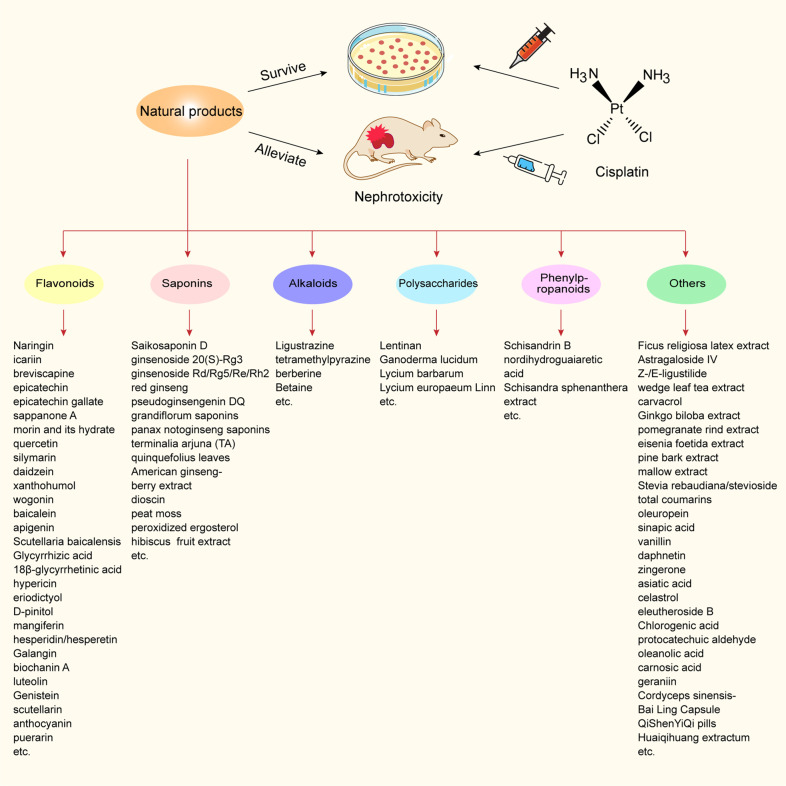
The summary of natural products to protect against cisplatin-induced nephrotoxicity. Potential natural product treatments for cisplatin-induced nephrotoxicity classified by chemical structures.

Traditional and complementary medicines, including a variety of natural products, such as herbs, vitamins, minerals, trace elements, and nutritional supplements, have been widely used in most countries [71]. Adopting natural products in healthcare can improve the physical fitness of patients. To better understand the roles of natural products in AKI, we summarized the protective effects of various classes of natural products on cisplatin-induced nephrotoxicity (Fig. 3 and Tables 2 and 3).

**Table 2:** Natural products in the treatments for cisplatin-induced nephrotoxicity classified by chemical structures.

Types	Natural products	Mechanisms/targets	Drug-drug interaction
1. Flavonoids	Naringin	Regulates redox balance, inhibits inflammatory, NF-κB activation, iNOS pathways, p53 activation, and apoptosis response [162]	Naringin, trimetazidine, or their combination could attenuate renal IR injury through inhibition of lipid peroxidase and enhancement of antioxidant activity [163]
	Icariin	Reduces oxidative stress, NF-κB activation, and inflammation cascade and apoptosis [75]	–
	Breviscapine	Inhibits oxidative stress: increases SOD and decreases MDA [76]	Icariin combined with breviscapine has synergistic effects on erectile function of spontaneously hypertensive rat [76]
	Epicatechin and epicatechin gallate	Inhibits oxidative stress, inflammation, NF-κB, NRF2/HO-1 signaling, reduces ERK activity, MAPK pathway [164]	Combined treatment of epigallocatechin gallate and Coenzyme Q10 attenuates cisplatin-induced nephrotoxicity via suppression of oxidative/nitrosative stress, inflammation, and cellular damage [165]
	Sappanone A	Reduces oxidative stress, upregulates NRF2 and HO-1, inhibits MPO, MDA, TNF-α, IL-1β, inhibits NF-κB activation [79]	–
	Morin and morin hydrate	Suppresses oxidative stress, inflammation and apoptosis, MAPK, PARP-1 regulation, inhibits autophagy stimulation [166]	–
	Quercetin	Inhibits oxidative stress, inflammatory and apoptosis response, MAPK signaling, inhibits M1, and upregulates M2 macrophage activities [81]	Quercetin-rich guava (*Psidium guajava*) juice in combination with trehalose reduces kidney injury of type II diabetic rats [167]; quercetin and allopurinol ameliorate kidney injury in STZ-treated rats [168]; combination of resveratrol and quercetin suppresses acetaminophen-induced AKI [169]; quercetin in combination with vitamins (C and E) improves renal injury in cadmium intoxicated rats [170]
	Silymarin	Selectively protects renal cells with no interfering effect on cancer cells [82]	Palmitoylethanolamide and silymarin combination attenuates the degree of renal inflammation in kidney ischemia and reperfusion model [171]
	Daidzein	Blocks inflammation, oxidative stress, and cell death, inhibits MAPK signaling pathway [83]	–
	Xanthohumol	Inhibits NF-κB, activates NRF2 signaling pathway [84]	–
	Wogonin	Inhibits RIPK1-mediated necrosis and attenuates WNT/β-catenin pathway, inhibits inflammation and apoptosis [172]	Baicalein, wogonin and oroxylin A combination contributes to anti-inflammatory effect [173]
	Baicalein	Upregulates antioxidant defense mechanisms and downregulates MAPKs and NF-κB signaling pathways [174]	–
	Apigenin	Suppresses oxidative stress and inflammation [86]	Apigenin enhances other antitumor drugs’ efficacy or reduces their toxicity in cancer treatments [175]
	*Scutellaria baicalensis* Georgi	Enhances tumor therapeutic efficacy and attenuates AKI [87]	Acacia catechu Willd and *Scutellaria baicalensis* Georgi combination suppress LPS-induced proinflammatory response [94]
	Glycyrrhizic acid and 18β-glycyrrhetinic acid	Inhibits NF-κB phosphorylation and HMGB1 cytoplasmic translocation, upregulates NRF2 and HO-1 [88]	–
	Hyperin	Inhibits NF-κB and upregulates NRF2 and HO-1 [89]	–
	Eriodictyol	Inhibits oxidative stress and inflammation, upregulates NRF2/HO-1 [90]	–
	D-pinitol	Inhibits inflammation, oxidative stress, MAPK pathway [176]	–
	Mangiferin	Upregulates NRF2 and activates PI3K, modulates MAPK pathway [177]	Mangiferin and morin combination attenuates oxidative stress [94]
	Hesperidin	Decreases oxidative stress, inflammation and DNA damage [178]	Taurine and hesperidin rescues carbon tetrachloride-triggered kidney damage in rats [178]
	Hesperetin	Inhibits oxidative stress, lipid peroxidation, inflammation and apoptosis, activates NRF2, inhibits MAPK signaling pathway [179]	Administration of naringenin and hesperetin combination downregulates FAK and p38 signaling pathways [131]
	Galangin	Inhibits ERK and NF-κB signaling, RIP1/RIP3-dependent necroptosis, oxidative stress, inflammation [180]	Quercetin and galangin combination enhances anti-inflammatory effect [181]
	Biochanin A	Inhibits inflammatory response and p53 apoptosis [182]	Formononetin and biochanin A modulate NF-κB/p-AKT signaling molecules [183]
	Luteolin	Decreases platinum accumulation and suppresses oxidative/nitrosative stress, inflammation and apoptosis [92]	–
	Genistein	Inhibits NF-κB and p53 activation [93]	–
	Scutellarin	Inhibits inflammation and apoptosis, activates autophagy [94]	Edaravone and scutellarin combination activates anti-inflammatory effect in ischemia injury [184]
	Anthocyanin	Inhibits TNF-α, IL-1β and increases BCL-2, antioxidant, antiapoptotic and anti-inflammatory responses [185]	–
	Puerarin	Inhibits TLR4/NF-κB signaling, promotes antitumor activity in COLO205 and HeLa [73]	The combination of tanshinone IIA and puerarin inhibits the immersion of inflammatory cells [186]
2. Saponins	Saikosaponin D	Reduces apoptosis, inhibits TNF-α, IL-1β and IL-6, and NO, reduces nitriding stress, and inhibits the activation of the NF-κB-P38-JNK-MAPK signaling cascades [97]	Antitumor effect is enhanced in combination saikosaponin D with SP600125 [187]
	Ginsenoside 20(S)-Rg3	Inhibits autophagy, blocks cell apoptosis, inhibits JNK-P53-caspase-3 signaling cascades [101]	Both studies of acute toxicity and seven-day repeated dose toxicity indicated the safety of the salvianolic acid B and ginsenoside Rg1 combination [188]
	Ginsenoside Rb3	Inhibits AMPK-/mTOR-mediated autophagy and apoptosis [102]	
	Ginsenoside Rd/Rg5/Re/Rh2, red ginseng, Pseudoginsengenin DQ, *Platycodon grandiflorum* saponins	Reduces oxidative stress, inflammation and apoptosis, reduces COX-2 and iNOS expression, sirt1/NF-κB and caspase signaling pathway, PI3K/AKT/Apoptosis signaling pathways [98, 99, 189–193]	
	*Panax notoginseng* saponins	Increases autophagy, BCL-2, reduces mitochondria-mediated endogenous apoptosis, HIF-1α/mitochondria/ROS pathway [194]	*Panax notoginseng* saponins could increase the gastrointestinal tract absorption of aspirin and salicylic acid [195]
	Saponins from *Terminalia arjuna*	Reduces oxidative stress, downregulates TGF-β, NF-κB and KIM-1 [100]	–
	Leaves of panax quinquefolius	Suppresses oxidative stress, inflammation and apoptosis, regulates PI3K/AKT/apoptosis	–
	American ginseng berry extract	Suppresses MAPK and NF-κB signaling pathways [196]	–
	Dioscin	Targets miR-34a/sirtuin 1 signaling pathway [103]	Dioscin reverses adriamycin-induced multidrug resistance by inhibition of the NF-κB signaling pathway [197]
	Peroxidized ergosterol	Reduces apoptosis, blocks MAPK-caspase-3 signaling cascade [104]	–
	Saponins extracted from the fruit of hibiscus	Blocks MAPKs signaling cascade [105]	–
3. Alkaloids	Ligustrazine	Inhibits oxidative stress, apoptosis, neutrophils infiltration and the overexpression of TNF-α and ICAM-1 [109]	Toxic study revealed ligustrazine was low toxic, LD_50_ was larger than 5 g/kg, both the level of ALT and AST and histopathology in the liver and kidney exhibited no distinctions between the tetramethylpyrazine, resveratrol, and curcumin (TRC) combination [198]
	Tetramethylpyrazine	Inhibits HMGB1/TLR4/NF-κB and activates NRF2 and PPAR-γ signaling pathways [110]	The effect of herbal compounds identified by network pharmacology approaches to reduce the toxicity of methotrexate was assessed by methotrexate-induced rat toxicity model [199]
	Berberine	Inhibits oxidative stress, inflammation, autophagy, and apoptosis [112]	Combination of berberine with pentoxifylline leads to more significant renoprotective effects than either berberine or pentoxifylline when used alone on diclofenac-induced AKI [200]; combination of berberine with doxorubicin is a novel strategy that has the potential for protecting against doxorubicin‑induced hepatorenal toxicity in clinical practice [201]
	Betaine	Alleviates inflammatory and apoptotic mediators, improves antioxidant abilities [113]	Caffeic acid phenethyl ester and betaine attenuate abamectin-induced hepatotoxicity and nephrotoxicity [181]
4. Polysaccharides	Lentinan	Activates NRF2-ARE, decreases ROS [117]	Lentinan combines with gemcitabine chemotherapy significantly inhibits UBC cell proliferation [202]
	*Ganoderma lucidum* polysaccharides/*Lycium barbarum* polysaccharides	Increases antioxidant enzymes, reduces oxidative stress and lipid peroxidation [118]	*Lycium barbarum* polysaccharides (LBP) and scopolamine combination prevents these SCO-induced reductions in cell proliferation and neuroblast differentiation [203]
	*Lycium europaeum* Linn	Antioxidant activities [119]	–
5. Phenylpropanoids	Schizandrin B	Inhibits oxidative stress, inflammatory and apoptosis response, β-catenin pathway, activates ERK/NF‑κB signaling [204]	Schizandrin B and lapatinib combination enhances the suppression on cell migration and invasion [205]
	Nordihydroguaiaretic acid	Inhibits oxidative stress, inflammatory and apoptosis response [121]	Erythropoietin (EPO) and nordihydroguaiaretic acid accelerate renal function recovery by stimulating tubular epithelial cell regeneration [206]
	*Schisandra sphenanthera* extract	NRF2 nuclear accumulation, inhibits ROS and increases GSH [122]	*Schisandra sphenanthera* extract could enhance the bioavailability of tacrolimus primarily through the inhibition of P-gp-mediated efflux and CYP3A-mediated metabolism in the intestine [207]
6. Others	*Ficus religiosa* latex extract	Antioxidant, increases GSH, SOD, CAT levels [111]	*Ficus religiosa* latex and constituents (glycoside, alkaloids, tannins, flavonoids, and amino acids) have excellent nephroprotective and curative activities [111]
	Astragaloside IV	Activates NRF2 and HO-1, inhibits NF-κB, induces autophagy, and limits NLRP3 expression [208]	–
	Z-ligustilide and E-ligustilide isolated from *Angelica sinensis*	Inhibits oxidative stress, suppresses β-catenin pathway [209]	–
	Wedge leaf tea extract	Inhibits oxidative stress, ROS, reduces apoptosis [140]	–
	Carvacrol	Suppresses oxidative stress, apoptosis, inflammation, suppresses ERK and PI3K/AKT pathways [124]	Combination of carvacrol and thymol upregulates the antimicrobial activity and antioxidant activity [210]
	*Ginkgo biloba* extract	Antioxidant activities, inhibits MDA, NO, MPO [211]	–
	Pomegranate rind extract	Inhibits oxidative stress, apoptosis, inflammation [143]	–
	*Eisenia foetida* extract	Prevents oxidative stress and inhibits lipid peroxidation [144]	–
	Pine bark extract	Increases antioxidant enzyme activities, inhibits lipid peroxidation [212]	–
	Mallow extract	Reduces MDA levels and inhibits inflammation [141]	–
	*Stevia rebaudiana*/stevioside	Inhibits ERK1/2, STAT3, and NF-κB [213]	–
	Total coumarins	Inhibits ERK1/2 and STAT3 signaling pathway, suppresses inflammation and apoptosis [134]	–
	Oleuropein	Inhibits ERK signaling, restores antioxidant system [214]	Oleuropein and 2-methoxyestradiol combination upregulates anticancer potential [215]
	Sinapic acid	Inhibits NF-κB and upregulates NRF2 and HO-1 [137]	–
	Vanillin	Inhibits NF-κB and decreases MDA, inhibits oxidative/nitrosative stress, inflammation and apoptosis [216]	Ortho-vanillin exacerbate the anti-arthritic effects of methotrexate in adjuvant-induced arthritis [217]
	Daphnetin	Inhibits NF-κB and upregulates NRF2 and HO-1 [138]	–
	Zingerone	Inhibits oxidative stress, apoptosis and inflammation [139]	Zingerone and dihydroartemisinin combination presents synergistic antimalarial activity [218]
	Asiatic acid	Suppresses IL-1β, TNF-α, MCP-1, and caspase-1 [135]	Combination of carnosine and asiatic acid enhances anti-inflammation activity [219]
	Celastrol	Inhibits NF-κB and improves mitochondrial function [136]	Combination of Lapatinib and celastrol downregulates subcellular distribution of HER2 [220]
	Eleutheroside B	Activates IGF pathway and reduces IGFBP-7 [125]	–
	Chlorogenic acid	Suppresses p53, activates caspase-3 and LC3-II expression, inhibits apoptosis and autophagy [126]	Combination of lapatinib with chlorogenic acid inhibits breast cancer metastasis by suppressing macrophage M2 polarization [221]
	Protocatechuic aldehyde	Suppresses NOX-mediated oxidative stress and inflammation [127]	–
	Oleanolic acid	Inhibits ERK, STAT3 and NF-κB, promotes sensitivity of Hela to cisplatin [222]	Combination of rho iso-alpha acids from hops, rosemary, and oleanolic acid decreased pain by 50% in patients with osteoarthritis [223]
	Green tea	Restores antioxidant defense system [224]	–
	Carnosic acid	Enhances SOD, CAT, GR, and GST activities, inhibits apoptosis [225]	Carnosic acid and fisetin combination therapy enhances inhibition of lung cancer through apoptosis induction [226]
	Emodin	Increases antioxidant enzyme activities, modulates AMPK/mTOR signaling pathways, activates autophagy [227]	Emodin combined with cytarabine induces apoptosis [228]
	Ethanolic extract of *Trigonella foenum-graecum*	Inhibits oxidative stress, apoptosis, inflammation [229]	Dietary fenugreek (*Trigonella foenum-graecum*) seeds and onion (*Allium cepa*) attenuate diabetic nephropathy [230]
	Geraniin	Inhibits NF-κB and upregulates NRF2 and HO-1 [231]	Geraniin combines with morphine or diclofenac to enhance anti-nociceptive effect [232]
	*Apodytes dimidiata*	Scavenges ROS, increases GSH, GPx, SOD, and catalase [233]	–
	Lycopene	Useful therapy for nephrotoxicity [234]	The combination therapy of rosmarinic acid and lycopene shows better protective effects than the corresponding monotherapy [235]
	Genipin	Inhibits oxidative stress, apoptosis, inflammation [236]	Combination of genipin and oxaliplatin enhances the therapeutic effects by upregulating BIM in colorectal cancer [237]
	*Schisandra chinensis* bee pollen extract	Inhibits oxidative stress, apoptosis, inflammation [238]	–
	*Schisandra chinensis* stems	Inhibits oxidative stress, apoptosis, inflammation [239]	–
	*Filipendula ulmaria* extract	Inhibits oxidative stress [240]	–
	Nigella sativa extract	Inhibits oxidative stress [241]	Mixed hydroalcoholic extracts of *Nigella sativa* and *Curcuma longa* improve adriamycin-induced renal injury in rat [242]
	Nigella sativa oil	Decreases BBM enzymes activities, inhibits oxidative stress [243]	Fish oil/*Nigella sative* volatile oil emulsion is the most promising hepato-regenerative and renoprotective formula [244]
	Danshen	Modulates NRF2 signaling pathway, inhibits oxidative stress [245]	The combination of rhein (RH) and danshensu (DSS) conferred a protective effect, as shown by a significant improvement in the chronic renal function [246]
	*Plantago major* extract	Inhibits oxidative stress [247]	*Plantago major* (300, 600, and 1200 mg/kg) and vitamin E significantly attenuated kidney tissue damage [248]
	Ethanolic fruit extract of *Citrullus colocynthis*	Inhibits oxidative stress [249]	–
	Ethanol leaf extract of *Andrographis paniculata*	Modulates NRF2/KIM-1 signaling pathway [250]	–
	*Lycium europaeum* methanol extract	Enhances antioxidant activities [251]	–
	*Porphyra yezoensis*	Inhibits MAPK/NF-κB pathways, inhibits inflammation [252]	–
	Whortleberry	Inhibits oxidative stress, caspase-3 level [128]	–
	Tangeretin	Inhibits oxidative stress and inflammation, regulates NF-κB-TNF-α/iNOS signaling pathway [253]	Combination of luteolin and tangeretin enhances anti-inflammatory activities [254]
	Cynaroside	Inhibits caspase-3/MST-1 signal pathway [129]	–
	Resveratrol	Inhibits oxidative stress, apoptosis and inflammation, activates ERK pathway, decreases cisplatin concentration, and lowers its accumulation [255]	Combination of hUCMSCs and resveratrol can better protect renal podocyte function, reduction of blood glucose and renal injury for diabetic nephropathy [256]; resveratrol (RES) and quercetin (QUR) can protect against APAP-induced nephrotoxicity [257]
	*Dendropanax morbifera*	Inhibits oxidative stress and inflammation [131]	–
	Troxerutin	Inhibits oxidative stress, inhibits MDA, increases SOD and GPx [132]	Troxerutin potentiates 5-fluorouracil treatment of human gastric cancer through suppressing STAT3/NF-κB and BCL-2 signaling pathways [258]
	Meclofenamic acid	Inhibits fat mass and obesity-associated protein (FTO)-mediated m6A abrogation [133]	Simvastatin in combination with meclofenamic acid inhibits the proliferation and migration of human prostate cancer PC-3 cells [259]
	QiShenYiQi (QSYQ) pills	Inhibits caspase-3, inhibits oxidative stress and apoptosis [146]	–
	Huaiqihuang (HQH) extractum	Reduces nuclear-cytoplasmic translocation of HMGB1 and inactivates TLR4 and NF-κB signaling pathway [145]	–
	Curcumin	Inhibits oxidative stress, apoptosis, inflammation, increases NAMPT and SIRT levels, inhibits M1 macrophage, and increases M2 macrophage polarization [260–262]	Curcumin and dexmedetomidine was effective in reducing oxidative stress and renal histopathologic injury in I/R rat model [261]; combination of curcumin and metformin might be functional to treat or inhibit nephrotoxicity [262]

**Table 3:** Natural products in the treatments for cisplatin-induced nephrotoxicity classified by mechanisms.

Mechanisms	Natural products types	Representative natural products
1. Cellular uptake and transport	Flavonoids	Formononetin
2. DNA damage	Flavonoids	Hesperidin
3. Apoptosis	Flavonoids	Naringin, Icariin, Morin and morin hydrate, Quercetin, Wogonin, Hesperetin, Luteolin, Scutellarin, Anthocyanin
	Saponins	Saikosaponin D, Ginsenoside 20(S)-Rg3, Ginsenoside Rb3, Ginsenoside Rd/Rg5/Re/Rh2, red ginseng, Pseudoginsengenin DQ, *Platycodon grandiflorum* saponins, Leaves of panax quinquefolius, Peroxidized ergosterol
	Alkaloids	Ligustrazine, Berberine, Betaine
	Phenylpropanoids	Schizandrin B, Nordihydroguaiaretic acid
	Others	Wedge leaf tea extract, Carvacrol, Pomegranate rind extract, Total coumarins, Vanillin, Zingerone, Chlorogenic acid, Carnosic acid, Genipin
4. Oxidative stress	Flavonoids	Icariin, Breviscapine, Epicatechin and epicatechin gallate, Sappanone A, Morin and morin hydrate, Quercetin, Daidzein, Baicalein, Apigenin, Eriodictyol, D-pinitol, Mangiferin, Hesperidin, Hesperetin, Galangin, Luteolin, Anthocyanin, etc.
	Saponins	Ginsenoside Rd/Rg5/Re/Rh2, red ginseng, Pseudoginsengenin DQ, *Platycodon grandiflorum* saponins, Saponins from *Terminalia arjuna*, leaves of panax quinquefolius, Dioscin, Saponins extracted from the fruit of hibiscus
	Alkaloids	Ligustrazine, Tetramethylpyrazine, *Ficus religiosa* latex extract, Berberine, Betaine
	Polysaccharides	Lentinan, *Ganoderma lucidum* polysaccharides /*Lycium barbarum* polysaccharides, *Lycium europaeum* Linn
	Phenylpropanoids	Schizandrin B, Nordihydroguaiaretic acid, *Schisandra sphenanthera* extract
	Others	Astragaloside IV, Z-ligustilide and E-ligustilide isolated from *Angelica sinensis*, Wedge leaf tea extract, Carvacrol, *Ginkgo biloba* extract, Pomegranate rind extract, *Eisenia foetida* extract, Pine bark extract, Mallow extract, Oleuropein, Sinapic acid, Vanillin, Zingerone, Protocatechuic aldehyde, Carnosic acid, Emodin, Apodytes dimidiate, Genipin
5. Inflammation	Flavonoids	Naringin, Icariin, Epicatechin and epicatechin gallate, Sappanone A, Morin and morin hydrate, Quercetin, Daidzein, Xanthohumol, Wogonin, Apigenin, Eriodictyol, D-pinitol, Hesperidin, Hesperetin, Galangin, Biochanin A, Luteolin, Genistein, Naringin, Scutellarin, Anthocyanin, Puerarin, etc.
	Saponins	Saikosaponin D, Ginsenoside Rd/Rg5/Re/Rh2, red ginseng, Pseudoginsengenin DQ, *Platycodon grandiflorum* saponins, Leaves of panax quinquefolius, American ginseng berry extract
	Alkaloids	Ligustrazine, Tetramethylpyrazine, Berberine, Betaine
	Phenylpropanoids	Schizandrin B, Nordihydroguaiaretic acid
	Others	Carvacrol, Pomegranate rind extract, Mallow extract, *Stevia rebaudiana*/stevioside, Total coumarins, Sinapic acid, Vanillin, Daphnetin, Zingerone, Asiatic acid, Celastrol, Protocatechuic aldehyde, Genipin
6. Autophagy	Flavonoids	Morin and morin hydrate, Scutellarin, Berberine
	Saponins	Ginsenoside 20(S)-Rg3, Ginsenoside Rb3, *Panax notoginseng* saponins
	Alkaloids	Berberine
	Others	Astragaloside IV, Chlorogenic acid, Emodin

### Flavonoids

Studies have shown that formononetin can effectively reduce OCT2 expression and increase MRP expression, resulting in decreased accumulation of cisplatin in renal tubular cells [72]. Similarly, puerarin protects against cisplatin-induced nephrotoxicity and promotes the antitumor activity of cisplatin in COLO205 and HeLa tumor cells in a dose-dependent manner [73]. Interestingly, naringin can alleviate cisplatin-induced renal dysfunction by inhibiting the inflammatory response and reducing apoptosis [74]. Flavonoids with multiple activities, such as icariin, breviscapine, epicatechin and epicatechin gallate, sappanone A, morin and its hydrate, quercetin, silymarin, daidzein, and xanthohumol, can reduce cisplatin-induced oxidative and nitrosative stress and decrease creatinine (Cre) and blood urea nitrogen (BUN) levels to improve renal function, thereby alleviating cisplatin-induced nephrotoxicity [75–84]. In addition, wogonin markedly inhibits receptor-interacting protein kinase 1-mediated necrosis and the canonical WNT pathway (WNT/β-catenin pathway) to protect against cisplatin-induced nephrotoxicity [85]. Further studies demonstrated that baicalein and apigenin ameliorated cisplatin-induced renal damage through the upregulation of antioxidant pathways and downregulation of the MAPK and NF-κB signaling pathways [86].

Interestingly, *Scutellaria baicalensis* Georgi not only enhances the therapeutic efficacy of cisplatin but also attenuates chemotherapy-induced AKI [87]. Glycyrrhizic acid, 18β-glycyrrhetinic acid, hypericin, and eriodictyol reduce AKI by inhibiting the cisplatin-induced phosphorylation of NF-κB and upregulating the expression of nuclear factor erythroid 2 (NFE2)-related factor 2 (NRF2) and HO-1 [88–90]. D-Pinitol and mangiferin attenuate inflammatory infiltration, DNA damage, and renal dysfunction in rats by modulating the MAPK pathway [91]. Furthermore, cisplatin-induced oxidative stress is mitigated by hesperidin and hesperetin by reducing MDA/Myeloperoxidase (MPO) levels and increasing SOD/Glutathione (GSH) levels. Galangin and the isoflavonoid biochanin A exhibit renoprotective effects in mice by targeting the inflammatory response and p53-mediated apoptosis. Importantly, luteolin significantly reduces histological and biochemical changes induced by cisplatin by blocking platinum accumulation and inflammation [92]. Genistein and naringin inhibit the NF-κB and iNOS pathways and p53 activation to improve HK-2 cell viability and kidney morphology in the presence of cisplatin and have become a potential effective treatment strategy for AKI [93]. A recent study demonstrated that scutellarin and anthocyanin from the fruits of *Panax ginseng* attenuate cisplatin-induced nephrotoxicity by inhibiting TNF-α [94]. In summary, flavonoids exhibit great potential as dietary supplements to ameliorate cisplatin-induced nephrotoxicity.

It is worth noting that the flavonoid phloretin is a robust toxicant (LC_50_ = 362 μM) that potentiates H_2_O_2_-induced toxicity, which is consistent with the previously noted cytotoxicity of phloretin and other hydroxychalcones. This toxicity is due to the oxidative activities of these polyphenols and the possible induction of mitochondrial toxicity [95].

Many flavonoids show strong protective effects against cisplatin-induced AKI. To date, researchers have found that many kinds of flavonoids activate NRF2/HO-1 signaling and inhibit NF-κB activity to alleviate kidney injury. More interestingly, some flavonoids not only protect against cisplatin-induced kidney injury but also synergistically inhibit the growth of tumors, enhancing the efficacy of cisplatin in tumor-bearing mice [74].  These results suggest that flavonoids may be used in the comprehensive treatment of cancer patients. Although flavonoids exhibit strong protection against kidney injury, there are some challenges in the clinical application of flavonoids. For example, monomers of flavonoid compounds are difficult to extract and have poor lipid solubility and low bioavailability, limiting their clinical applications [96]. If researchers can overcome these challenges, flavonoids will become promising drugs for AKI treatment.

### Saponins

Oxidative stress and inflammation are important mechanisms involved in the pathogenesis of AKI. Some studies have shown that saikosaponin D can increase the survival rate of HK-2 cells and maintain the normal morphology of the nucleus. Saikosaponin D can inhibit the activation of the NF-κB-P38-JNK-MAPK signaling cascade, thereby reducing cisplatin-induced apoptosis [97]. Red ginseng, ginsenoside Rg5, and *Platycodon grandiflorum* saponins can inhibit inflammation by reducing the expression of cyclooxygenase-2 and iNOS to inhibit acute tubular necrosis and apoptosis [98, 99]. Renal oxidative stress, as evidenced by increased MDA levels and declines in GSH and SOD activities, is significantly reduced by saponins from *Terminalia arjuna* [100].

In addition, some saponin components mainly regulate autophagy and apoptosis to exert protective effects against kidney injury. Ginsenoside 20(S)-Rg3 and ginsenoside Rb3 can inhibit autophagy to improve renal injury by blocking the JNK-P53-caspase-3 signaling cascade [101, 102]. *Panax notoginseng* saponins can improve cisplatin-induced damage to mitochondria, reduce mitochondria-mediated endogenous apoptosis, and enhance autophagy in renal cells, thus reducing cisplatin-induced nephrotoxicity. Dual luciferase reporter assays and molecular docking assays demonstrated that dioscin could target the miR-34a/sirtuin 1 signaling pathway to alter cisplatin-induced nephrotoxicity [103]. Peat moss sphagnum palustre can prevent colon cancer and has antibacterial effects, and peroxidized ergosterol can reduce cisplatin-induced apoptosis and improve cisplatin-induced kidney injury [104]. Other researchers have found that saponins extracted from Hibiscus fruit have protective effects on cisplatin-induced cytotoxicity in LLC-PK1 kidney cells [105].

Compared with other saponin components, ginsenoside 20(S)-Rg3 and Rb3 inhibit autophagy to block apoptosis [101, 102]. Further studies are needed to examine whether all saponin components can play important roles in regulating autophagy to protect against AKI. On the other hand, some studies show that saponin components play protective roles in alleviating kidney injury by regulating the NF-κB signaling pathway to reduce inflammation. However, whether saponin components affect the recruitment of immune cells and which type of immune cell is the main regulator are unclear. More studies need to be conducted to elucidate the role of immune cells in saponin component-mediated inhibition of the inflammatory response to protect against cisplatin-induced nephrotoxicity.

In summary, these findings clearly suggest that saponin components can exert protective effects against cisplatin-induced nephrotoxicity, mainly due to the regulation of autophagy and inhibition of oxidative stress, inflammation, and apoptosis. However, both in vitro and in vivo studies have demonstrated that *M. charantia* may also exert toxic or adverse effects under different conditions and can decrease plasma progesterone and estrogen levels in a dose-dependent manner [106]. This plant causes acute symptoms such as changes in respiratory and heart rates and may induce termination of early pregnancy and cause abortion [107]. In addition, it has been reported that *M. charantia* fruit causes abdominal pain and diarrhea in individuals with diabetes [108].

### Alkaloids

Studies have shown that ligustrazine can reduce the levels of urinary protein, as well as serum Cre and BUN, and enhance the antioxidant capacity, thus exerting a certain protective effect against nephrotoxicity [109]. Tetramethylpyrazine inhibits HMGB1/TLR4/NF-κB and activates the NRF2 and PPAR-γ signaling pathways to achieve nephroprotective effects [110]. *Ficus religiosa* latex extract has glycoside, alkaloid, and amino acid constituents and shows excellent nephroprotective and curative effects in rats [111]. A study indicated that berberine exerted a nephroprotective effect via the inhibition of oxidative stress, inflammation, autophagy, and apoptosis in cisplatin-induced AKI [112]. Betaine exerts renoprotective effects by alleviating inflammatory and apoptotic mediators and improving antioxidant capacity in rats and may be a beneficial dietary supplement to attenuate cisplatin-induced nephrotoxicity [113].

In contrast, alkaloids found in aconitum species are highly toxic cardiotoxins and neurotoxins [114]. Moreover, further investigations are necessary to determine the exact toxicological mechanisms because the coadministration of alkaloids with drugs that are substrates of DMEs and/or ETs may cause herb-drug interactions [115]. In addition, dehydropyrrolizidine alkaloid (DHPA) can induce chronic disease, which may accumulate over a long period of time and develop slowly until liver failure. The incidence of tumors in rodents increased even in response to very low DHPA doses for a short period of time [116]. These concerns have limited animal or human exposure to alkaloids.

Taken together, these studies demonstrated that alkaloids could ameliorate cisplatin-induced AKI in mice and rats. Alkaloid treatment regulates immune cell infiltration, inhibits oxidative stress, and suppresses apoptosis in the kidney to protect against cisplatin-induced AKI. Mechanistically, some alkaloids efficiently reverse the cisplatin-induced activation of the TLR4/NF-κB pathway and ameliorate renal oxidative stress by increasing GSH, SOD, and CAT levels. Further investigations aimed at delineating the signaling pathways involved in the beneficial effects of alkaloids on cisplatin-induced AKI are needed.

### Polysaccharides

Lentinan can alleviate cisplatin-induced apoptosis of HK-2 human kidney proximal tubular cells and disrupt renal function in mice. The mechanism is related to the activation of the NRF2-ARE signaling pathway and decrease in intracellular ROS. Moreover, lentinan also inhibits the proliferation of HeLa and A549 cells [117]. In addition, *Ganoderma lucidum* polysaccharides and *Lycium barbarum* polysaccharides can increase the activities of antioxidant enzymes and reduce the levels of oxidative stress and lipid peroxidation, thereby protecting against cisplatin-induced nephrotoxicity [118]. *Lycium europaeum* Linn is a well-known medicinal plant and is a source of polysaccharides with antioxidant activities in vivo and in vitro [119].

No adverse reactions to polysaccharides have been reported so far, but there can be slight gastrointestinal reactions, which can be alleviated after 1 week of administration. In addition, astragal polysaccharides may cause dry mouth, chest distension, easy excitation, and other adverse reactions. No recommendations on the clinical use of polysaccharides are currently available. In summary, polysaccharide components generally show obvious antioxidant activities by decreasing ROS levels and increasing antioxidative enzymes. However, it is still unknown how polysaccharide components exhibit oxidative effects to protect against kidney injury.

### Phenylpropanoids

Schizandrin B and nordihydroguaiaretic acid have inhibitory effects against cisplatin-induced nephrotoxicity, and their renoprotective mechanisms are associated with oxidative stress, inflammatory response, and apoptosis [120, 121]. It is believed that *Schisandra sphenanthera* extract facilitates the nuclear accumulation of the transcription factor NRF2 to mitigate cisplatin-induced nephrotoxicity, which is important for therapeutic approaches to AKI [122]. In some cases of illness, star anise tea can cause severe neurological and gastrointestinal toxicity, which is characterized by convulsions, diarrhea, and vomiting [123]. No recommendations on the clinical use of *Schisandra sphenanthera* extract are currently available.

Overall, phenylpropanoids inhibit oxidative stress, inflammation, and apoptosis to alleviate AKI by increasing GSH levels and NF-κB signaling. Phenylpropanoids mainly play roles of preventing or protecting against cisplatin-induced kidney injury through these three mechanisms, but the interactions between these mechanisms are still unclear.

### Others

Current evidence suggests that carvacrol attenuates AKI by suppressing oxidative stress, apoptosis, and inflammation by modulating the extracellular-regulated protein kinases (ERK) and PI3K/AKT pathways [124]. In addition, eleutheroside B activates the insulin-like growth factor pathway and reduces the expression of insulin-like growth factor binding protein 7, thereby increasing HK-2 cell viability against cisplatin-induced damage [125]. Chlorogenic acid significantly suppresses the expression of p53, active caspase-3 and light chain 3-II, suggesting the inhibition of both apoptosis and autophagy [126]. Notably, protocatechuic aldehyde blocks cisplatin-induced AKI by suppressing Nox-mediated oxidative stress and inflammation without affecting the antitumor activity of cisplatin [127]. In addition, whortleberry, tangeretin, cynaroside, resveratrol, *Dendropanax morbifera*, troxerutin, and meclofenamic acid also have certain inhibitory effects on nephrotoxicity [128–133].

A variety of other natural products show renoprotective effects mainly by regulating the NF-κB pathway. For example, astragaloside IV effectively protects against cisplatin-induced nephrotoxicity by activating NRF2 and HO-1 and inhibiting the NF-κB pathway. The natural sweetener *Stevia rebaudiana* and its constituent stevioside protect against cisplatin-induced nephrotoxicity by inhibiting ERK1/2, STAT3, and NF-κB activation, as do total coumarins from *Hydrangea paniculata* [134]. Increasing evidence suggests that asiatic acid, a terpene, suppresses the increased mRNA expression of the proinflammatory cytokines interleukin-1β, NF-κB, and MCP-1 in the kidneys [135]. Moreover, celastrol can ameliorate cisplatin-induced nephrotoxicity by inhibiting NF-κB and improving mitochondrial function [136]. Oleuropein, sinapic acid, vanillin, daphnetin, and zingerone can inhibit NF-κB activation and upregulate the expression of NRF2 and HO-1 to prevent cisplatin-induced nephrotoxicity [137–139].

In addition, extracts from various natural plants can play protective roles in cisplatin-induced nephrotoxicity. Wedge leaf tea extract is used in Mongolian medicine to treat urinary system-related diseases by inhibiting renal oxidative stress and reducing apoptosis caused by cisplatin [140]. Mallow extract can reduce MDA levels and inhibit the release of inflammatory factors, thus improving the understanding of kidney protection [141]. Moreover, *Ginkgo biloba* extract, pomegranate rind extract, and *Eisenia foetida* extract are rich in flavonoids and terpenoid lactones, which can remove excess free radicals and inhibit lipid peroxidation, thus resisting cisplatin-induced nephrotoxicity [142–144]. A recent study clarifies that Huaiqihuang extract, a kind of Chinese herbal complex, reduces the nuclear-cytoplasmic translocation of HMGB1 and inactivates the TLR4 and NF-κB signaling pathways, exerting robust renoprotective effects [145].

By replenishing spirits and activating blood circulation, some traditional Chinese medicines and compound preparations can attenuate cisplatin-induced nephrotoxicity. For example, both artificial *Cordyceps sinensis*-Bailing capsules and QiShenYiQi pills, which are compound Chinese medicines, can inhibit the expression of caspase-3 in kidney tissue, thereby protecting the kidneys [146]. In addition, Astragalus injection can prevent kidney morphological and functional damage caused by cisplatin without reducing its antitumor activity. Astragalus contains saponins, polysaccharides, flavonoids, trace elements, and amino acids, which can scavenge free radicals to regulate immune functions [147].

Our review suggests that numerous natural products that possess potent medicinal properties, such as flavonoids with antioxidant and anti-inflammatory properties, are capable of protecting against kidney injury based on various promising laboratory findings. The factors can be applied as supplementary regimens or combinations against cisplatin-induced nephrotoxicity. Many active compounds have attracted much attention from chemists. Through structural modifications and optimization, the synthesis of compounds with improved activity and biosafety is the ultimate goal of every study. However, at present, many monomers with good activity are obtained from complicated sources, and the isolation and extraction processes have not been perfected [148]. Therefore, it is difficult to thoroughly isolate these compounds, the purity cannot be guaranteed, and the compounds have a variety of functions. In addition, some compounds may have multiple targets; thus, their effects may be multifaceted, including protective effects and side effects.

In addition to focusing on compounds in natural plants, some active ingredients in the ocean have been less well studied. The ocean is not only a huge treasure trove of materials but also a source of natural medicines with great potential. Some marine drugs have strong antioxidant effects, pharmacological effects, and clinical applications, such as seaweed, laminaria, oyster, cuttlefish bones, and wakame [149]. The overfishing of marine resources and other reasons are the reasons that natural resources cannot meet increasing needs. Therefore, we should focus our attention on the development and utilization of traditional Chinese patent medicines and traditional Chinese medicinal materials. With marine medicine sources, the scientific formula should be strengthened to make new doses and forms easy to use. For example, a soft capsule of Huoxiangzhengqi liquid mixed with a marine medicine source is a very successful example.

## Future perspectives

Worldwide, AKI is a serious health problem, and the number of cases is increasing because of the side effects of medications or the complications of other diseases. In addition, the onset of AKI leads to chronic kidney disease, uremia, and hypertensive nephropathy [150–152]. The pathogenesis of cisplatin-induced nephrotoxicity is complex, early diagnosis is difficult, and effective treatment options are lacking. The urgency of developing a renoprotective strategy has pushed researchers to look at active natural products with few side effects. There are broad development prospects in the treatment of cisplatin-induced nephrotoxicity compared to the existing relief pathways. For example, curcumin has been used in traditional medicine because of its efficacy against kidney damage [153–156]. Recent reports show that pretreatment with curcumin can ameliorate cisplatin-induced kidney damage by suppressing inflammation and apoptosis [157].

The mechanisms of cisplatin-induced kidney damage involve various pathways, such as inflammatory mediators, oxidative stress, necrosis and apoptosis, and autophagy. To date, researchers have not found that these mechanisms are involved in cisplatin-induced nephrotoxicity, starting with excess ROS generation, which leads to oxidative stress, triggering inflammatory and autophagy pathways that damage DNA and induce apoptosis in the kidney. It is still unclear how the various pathways integrate and ultimately lead to kidney damage. In recent years, many natural products have been discovered by different mechanisms. A natural compound may have multiple active targets rather than only one unique target. Therefore, a natural product may play multiple roles and exhibit wide use and may have increased potential toxicity or side effects. Since some pathways of cisplatin-induced kidney injury are also involved in the antitumor effects of cisplatin, natural products may also affect cisplatin-mediated antitumor effects. While most compounds have anti-inflammatory and antioxidant properties, NAC and vitamin E have been reported to act as antioxidants and contribute to the development of lung cancer [158]. Therefore, it is unclear whether natural compounds with antioxidant activity interfere with the development of tumors while protecting against kidney injury. In this case, in addition to check the protective role against AKI, it is necessary to further study if natural products have effects on tumor growth, which may help to break through the limited use of cisplatin in clinic.

It is worth noting that natural products that have robust therapeutic effects on cisplatin-induced AKI also alleviate kidney diseases caused by other factors. Further research is needed to verify the beneficial effects of certain products on humans and other animals with kidney diseases to elucidate the detailed mechanisms of the renoprotective effects. To achieve the desired protective effect against nephrotoxicity, researchers should take all aspects of the relevant mechanisms into account and consider comprehensive measures or combinations of drugs. In addition, although certain natural products are excellent in protecting against kidney damage in vitro and in vivo, it is necessary to study the optimal dose for protecting against different tumors and different cisplatin strengths.

Furthermore, the development of molecular biology technology has led to the research of targeted therapy using cisplatin and natural products or derivatives that are highly selective for the kidney or tumor as carriers, and chemically coupling these factors into biological treatments. Direct delivery of cisplatin to the tumor site rather than the kidney can not only reduce the amount of cisplatin needed but also improve the efficacy and reduce adverse reactions. This opens up new ideas for the study of protective measures against cisplatin-induced nephrotoxicity.
